# Integrating a Hive Triangle Pattern with Subpixel Analysis for Noncontact Measurement of Structural Dynamic Response by Using a Novel Image Processing Scheme

**DOI:** 10.1155/2014/375210

**Published:** 2014-05-11

**Authors:** Yung-Chi Lu, Shih-Lin Hung, Tzu-Hsuan Lin

**Affiliations:** ^1^Department of Civil Engineering, National Chiao-Tung University, 1001 University Road, Hsinchu 30010, Taiwan; ^2^Sinotech Engineering Consultants Inc., 171 Section 5, Nanking East Road, Taipei 10569, Taiwan

## Abstract

This work presents a digital image processing approach with a unique hive triangle pattern by integrating subpixel analysis for noncontact measurement of structural dynamic response data. Feasibility of proposed approach is demonstrated based on numerical simulation of a photography experiment. According to those results, the measured time-history displacement of simulated image correlates well with the numerical solution. A small three-story frame is then mounted on a small shaker table, and a linear variation differential transformation (LVDT) is set on the second floor. Experimental results indicate that the relative error between data from LVDT and analyzed data from digital image correlation is below 0.007%, 0.0205 in terms of frequency and displacement, respectively. Additionally, the appropriate image block affects the estimation accuracy of the measurement system. Importantly, the proposed approach for evaluating pattern center and size is highly promising for use in assigning the adaptive block for a digital image correlation method.

## 1. Introduction

Structural health monitoring (SHM) detects a damage and characterization strategy for engineering structures. Conventional SHM technology collects structural static or dynamic response data by using wire-based systems. However, installing these wire-based systems can be expensive in labor, cost, and time [1]. To overcome these obstacles, several technologies have emerged to reduce the considerable efforts associated with devising a measurement system. These technologies include optical, laser, ultrasound, and wireless. As a mounted wireless module with a sensing device, a wireless sensing device simplifies the installation process and significantly reduces installation costs. Although measurement data are gained through radio waves, packets losses and power usage must be addressed [2]. Given the rapid advances in optical imaging hardware technology, the use of digital photography in structural monitoring systems has received considerable interest among researchers. Digital image analysis technology is adopted in many disciplines, especially in the recent decade [3–9]. Peters and Ranson [3] proposed digital image schemes to measure surface displacement in mechanical engineering. Laser speckle metrology can be described in terms of the undeformed and deformed coordinates of a body. Chu et al. [4] cited major limitations of speckle schemes: stability requirements and the laborious and time consuming nature of data processing. As a theory used in experimental mechanics, the digital image-correlation (DIC) method is a highly accurate means of determining rigid-body translations and rotations. Pan and Wang [5] proposed using a camera and a transmission diffraction grating to measure the surface profile and deformation of small-scale objects. Amodio et al. [6] examined the feasibility of constructing a short time, real-time speckle correlation device, while considering the availability of high resolution cameras and high-speed processors at competitive retail prices. Their method can be viewed as an extension to digital images of the conventional white light speckle photography approach. The modified algorithm can process hundreds of pictures in an acceptable time. Chung et al. [7] demonstrated the proof-of-concept in which emerging digital image processing techniques allow for system identification nonintrusively and remotely. The digital imaging procedure and inverse analysis algorithms developed for the friction problem can be extended to identify nonlinear mechanical and structural characteristics. Shih et al. [8, 9] developed the digital image correlation (DIC) method for determining the surface smoothness of construction materials and to monitor the dynamic responses of buildings under earthquake excitation. The accuracy of the DIC method is sufficiently high for several applications.

Digital image analysis technology is characterized by an enormous amount of data. In a particular time point, using wireless or wired measurement methods can obtain one data set, implying the structural dynamic response of a sensor. However, digital image analysis technology must acquire an image that consists of a specific pattern and leave a sufficient amount of displacement to ensure that the specific pattern is displayed in the acquired image at different time point. The amount of data of the acquired image may be thousands of times or more than that of wireless or wired measurement methods. Data format of the wired measurement method is assumed here to be a single-precision floating point. When the sampling rate is 200, the data storage capacity is 800 bytes/s. The acquired image size is assumed here to be 1024 × 50, and one grayscale pixel is stored with 8 bits, subsequently allowing for the recording of 256 different intensities. In an identical sampling rate, the image storage capacity is 10,240,000 bytes equivalent to 12,800 times the data storage capacity. The acquired image that is the full image of a camera has even greater variations. Therefore, in this work, a region of interest selected from the full image of camera is saved as the acquired image. The region may consist of a specific pattern or more patterns in a different area of the images. The specific pattern is generally painted on a different floor for the measured structure. The region covers all patterns on each floor of the structure.

An image block is a subset of an acquired image. An acquired image which is undisturbed by an external load is assigned as the source image. As a subset of the source image, a source image block consists of a specific pattern with a significant contrast. Additionally, an acquired image disturbed by an external load is assigned as the target image. In a target image, several target image blocks can be assigned in many positions. Dynamic displacement of the specific position can be estimated using an adaptive target image block to compare with a source image block in order to calculate its cross-correlation. The size and location of an image block affect the accuracy and precision of a digital image correlation algorithm. Other influential factors include inherent image noise and subpixel interpolation approximation [10]. Digital image correlation refers to a class of nondestructive methods that acquire images of an object, store images in digital form, and perform image analysis to extract a full-field shape and deformation and/or motion measurements [11].

A higher accuracy and precision generally require a larger image block size. While an adaptive pattern is painted on the surface of a structure, the displacement of a higher accuracy and precision can be estimated by digital image analysis technology and the image block just filled with a specified pattern. The specified pattern can provide a significant contrast in the acquired images to enhance the effect of digital image correlation coefficient on the acquired images. When the acquired region of image is enlarged, digital image analysis technology can estimate the dynamic response over a wider range. Owing to the enormous amount of data from the acquired image, the transmission rate must be sufficiently large. Otherwise, the acquired images may lose some frame in the image capture process, and the image missing rate becomes more severe with a higher sampling rate. To reduce the image missing rate, the acquired range of an image is reduced to the region of interest. Additionally, the proposed measurement system records the missing number of acquired images to compensate the missing image with an interpolation method in postprocessing.

The optical image facility has advanced annually. The resolution of an acquired image can be enlarged by using a telescope lens, and a high frequency response of structure can be evaluated by a high-speed digital camera. However, image exposure time is reduced in the high-speed image capture process, implying that the acquired image may be too dark to identify; in addition, the processing time of acquiring an image may be too short to retrieve all images in every time interval. Therefore, a smaller and adaptive image is of priority concern. In this work, a hive triangle pattern (HTP) is stuck on a small three-story frame, and an evaluation method of the pattern center and size can register the location accurately and estimate the pattern size. The pattern increases the efficiency of a correlation coefficient. Analysis results indicate that the relative error is only slight, and the evaluated displacement time history closely resembles the numerical solution and LVDT.

## 2. Research Problem and Experimental Setup

**Figure 1:**
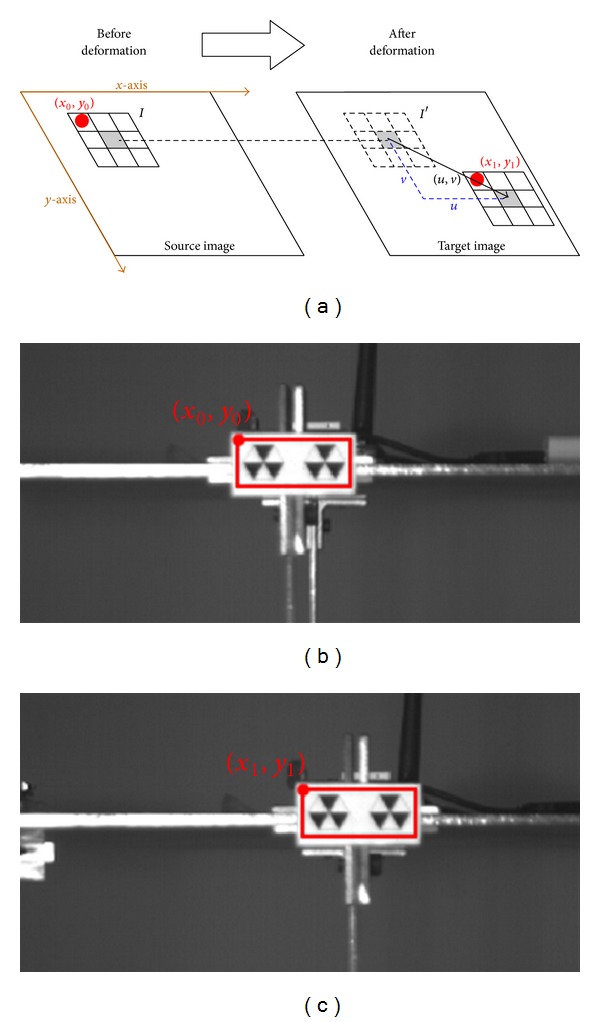
(a) Simplified diagram of source and target image, showing displacement variation of image block. (b) Source images. (c) Target image.

This work introduces a digital image displacement measurement problem under a source and target image. The object of a source image may appear somewhere in a target image. When the object is a rigid body, the displacement may also include the body rotations. However, the displacement of *y*-axis in relation to the *x*-axis is negligible. Therefore, the experimental result can be regarded as a linear translation. The simplified form of displacement estimation is expressed as follows:

(1) $$\begin{array}{c} \text{maxCC}\left(I_{x_{0},y_{0}},I_{x_{1},y_{1}}^{\prime }\right), \end{array}$$

where *I* denotes the based block from source image *S*, *I*′ represents the estimated block from target image *T*, the subscripts denote the coordinate *x* and *y* correspondence *S* and *T*, and CC refers to the correlation coefficient function to evaluate with the two blocks. The coordinate (*x*_0_, *y*_0_) of a source image is predefined with an appropriate value described later, and the coordinate (*x*_1_, *y*_1_) of a target image is selected from a measurement system to obtain the maximal correlation coefficient.  Figure 1 shows these parameters and actual images.

**Figure 2:**
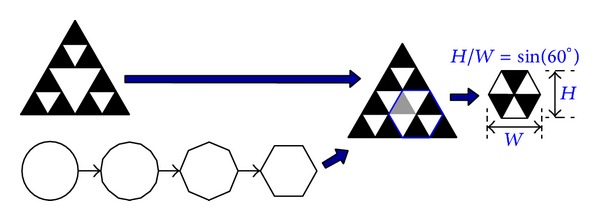
Generation of hive triangle pattern from circle and Sierpinski triangle.

For two-image block, the value of the correlation coefficient represents the degree of their similarity to each other. Theoretically, the object coordinate of source image changes to another location appearing on the target image in which the maximal correlation coefficient should be one, indicating their identical coordinates. However, the images shootings at different time points always differ from each other, even if the object of an image remains constant. Although the maximal correlation coefficient is always less than one, the estimation scheme can determine the accuracy displacement between images based on the similarity. A specified pattern which is painted on a structure can provide the necessary contrast to correlate images well to enhance the effect of the correlation coefficient on image similarity. In this work, the specified pattern is a hive triangle pattern which is derived from circle and Sierpinski triangle, as shown in Figure 2.

Selecting an image block is of priority concern when using a correlation coefficient to estimate object displacement. As location and size are two major factors for the image block, this work estimates efficiently the appropriate value of the factor with the proposed evaluation method by using the hive triangle pattern. The hive triangle pattern has the black and white characteristics. The pattern causes the image pixel values to significantly differ in the evaluated image. Despite the very small displacement of an object, the value of correlation coefficient can still clearly show how the images differ from each other.

**Figure 3:**
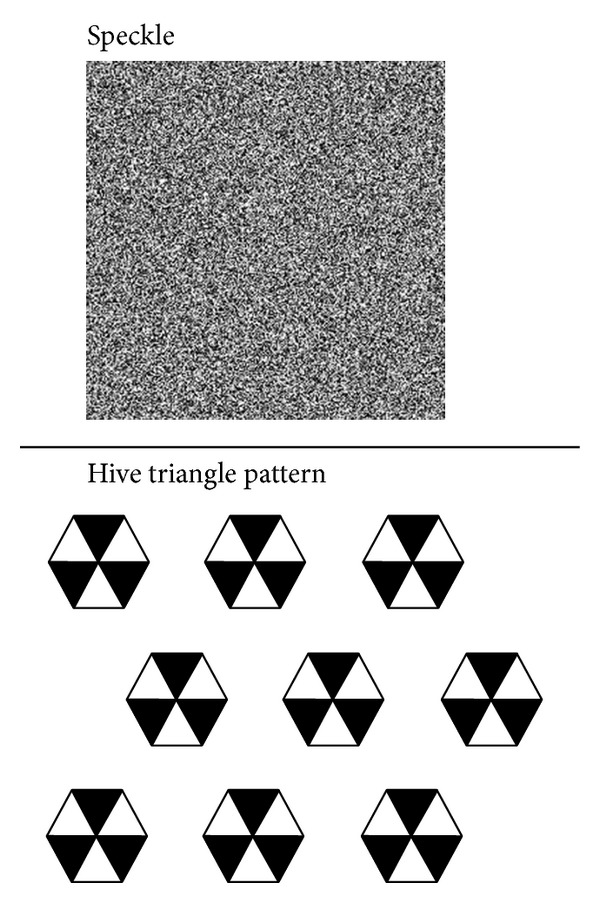
An image frame that includes the speckle and hive triangle patterns is used as reference object in numerical simulation.

Numerical simulation of a photography experiment at a short range demonstrates the feasibility of the proposed approach. The speckle and hive triangle patterns are combined as a single image frame, and a Flash animation program displays the frame in different locations with sine variation.  Figure 3 shows the speckle and hive triangle patterns.

**Figure 4:**
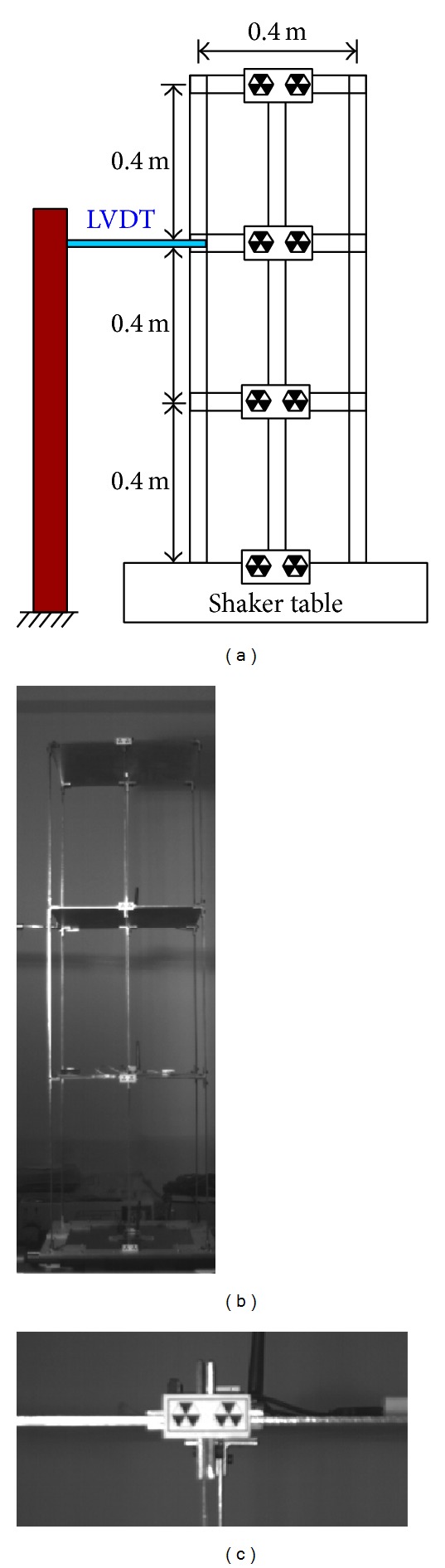
Configuration of small three-story frame and an LVDT on second floor (a). Image frame captured using measurement system with lower (b) and higher resolution (c).

In another experiment, a small three-story frame is mounted on a small shaker table and an LVDT is set on the second floor (Figure 4). To compare those results with those of LVDT, the experiment focuses on the second floor, as shown in the bottom image of Figure 1.

Hardware of the digital image measurement system consists of a high-speed digital camera (Basler A504kc, sampling rate of 500 Hz), camera lens, and high level computer with an image acquisition card. The digital camera is a color model whose image-type is Bayer pattern; decoding must be performed to obtain acceptable gray images. The high level computer is equipped with an i-RAM expansion card for increasing the access speed. The expansion card has the performance benefits of solid state storage; however, a maximum capacity of 4 GB limits the number of image frames. Fortunately, the image storage capacity of most of the experiment is less than 4 GB. However, the processing and access speed is still too slow, and the acquisition data lose some frames in a higher sampling rate. Therefore, the lost data is interpolated linearly as compensation.

## 3. Methods

**Figure 5:**
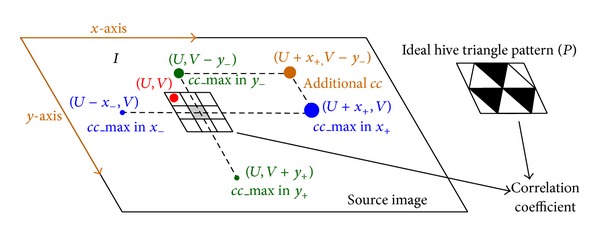
Correct location of pattern is estimated from source image and ideal hive triangle pattern with digital image correlation coefficient. Three largest circles (green, blue, and orange) indicate three largest correlation coefficients; the highest correlation coefficient is in *x*_+_ direction.

The work presents a novel hive triangle pattern to paint on the surface of structure. The pattern location affects the displacement estimation accuracy.  Figure 5 describes a scheme to estimate the original location (*x*_0_, *y*_0_) of the hive triangle pattern. Two acquired images are first selected as *S* and *T*. The image *S* is an undeformed image, and the image *T* is a slightly deformed image relative to image *S*. A simple difference method is incorporated in the two images, as shown in (2). The equation value indicates the maximal difference (255) and is exactly identical (0) of specified pixel. The pixel value of the same background is close to zero, and the value of pattern position is relatively large:

(2) $$\begin{array}{c} D=\left|S-T\right|. \end{array}$$

Additionally, the approximate coordinates of the pattern as in (3) are located using a mean-max method. The method calculates the means of all columns and rows in the difference image. The maximum value of the mean averages of the columns and rows indicates the *x*-axis and *y*-axis coordinate, respectively:

(3) $$\begin{array}{c} \bar{X_{i}}=\frac{\sum_{j=1}^{n}D\left(i,j\right)}{n},\;\;\bar{Y_{j}}=\frac{\sum_{i=1}^{m}D\left(i,j\right)}{m},\\ \tilde{X}=U,\;\text{where}\;\;\bar{X_{U}}>\bar{X_{i}}\;\;\text{for}\;\;i=1\sim m\;\;\text{and}\;\;i\neq U,\\ \tilde{Y}=V,\;\text{where}\;\;\bar{X_{V}}>\bar{X_{j}}\;\;\text{for}\;\;j=1\sim n\;\;\text{and}\;\;j\neq V. \end{array}$$

Moreover, the algorithm creates an ideal hive triangle pattern (*P*) for using to calculate the correlation coefficient with the source image. The correlation coefficient has many formulations. This work uses the following equation:

(4) $$\begin{array}{c} C=\frac{\sum \sum \left[\left(f-\langle f\rangle \right)\cdot \left(g-\langle g\rangle \right)\right]}{{\left[\sum \sum {\left(f-\langle f\rangle \right)}^{2}\cdot \sum \sum {\left(g-\langle g\rangle \right)}^{2}\right]}^{1/2}}. \end{array}$$

In (4), *f* and *g* are image blocks that represent the pixel value of the source image and target image, respectively. The sign 〈·〉 denotes the mean operator. Precision of the measurement unit is an integer pixel value with original images. The subpixel analysis can improve the precision based on the subpixel estimation of a target image.

Finally, a bilateral search can determine the maximal correlation coefficient of an individual direction. An additional point is determined by the previous maximal correlation coefficients. For instance, the maximal correlation coefficient of *X*-axis appears to be in a positive direction (right), and the maximal correlation coefficient of *Y*-axis appears to be in a negative one (up). The additional point is assigned the upright location (Figure 5). Notably, the correlation coefficient of the new point may be less than the previous maximal correlation coefficient. Therefore, the three larger correlation coefficients are evaluated simultaneously to determine the adaptive moving step.  Figure 5 illustrates the three larger correlation coefficients in the search mechanism, and the cc_max of *x*_+_ direction is the largest correlation coefficient. The initial location (*U*, *V*) moves to the new location (*U* + *x*_+_, *V*) in the next iteration. In the iterative process of the proposed approach, the final maximal correlation coefficient is determined and the evaluated location of pattern is found. The method may fail to locate the position of the hive triangle pattern due to an inappropriate target image. Experimental results indicate that the maximum correlation coefficient is higher than 0.95 in the correct search and lower than 0.75 in the fail search. Therefore, an appropriate threshold can help to determine the validity of the search. In the fail situation, another target image is selected to evaluate the maximum correlation coefficient again.

**Figure 6:**
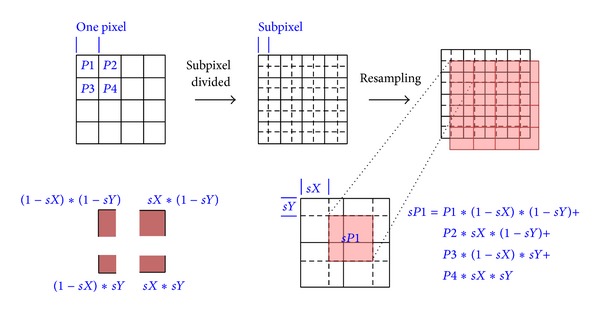
Subpixel and formula (sP1) for estimating its gray level. For block that must be resample owing to subpixel displacement, gray level of every pixel must be recalculated using estimation formula.

Another simple algorithm based on the pattern location (*x*_0_, *y*_0_) evaluates the pattern size. Owing to the triangle characteristic of the hive triangle pattern, its height has a fixed ratio (sin 60°) with the width. The maximum correlation coefficient can be evaluated based on the source image block and ideal pattern with different sizes. The pattern width (*W*) is determined in terms of integer-pixel accuracy. In this work, the pattern size of subpixel precision is estimated using a subpixel technician.  Figure 6 shows the estimation formulation of the subpixel.

**Figure 7:**
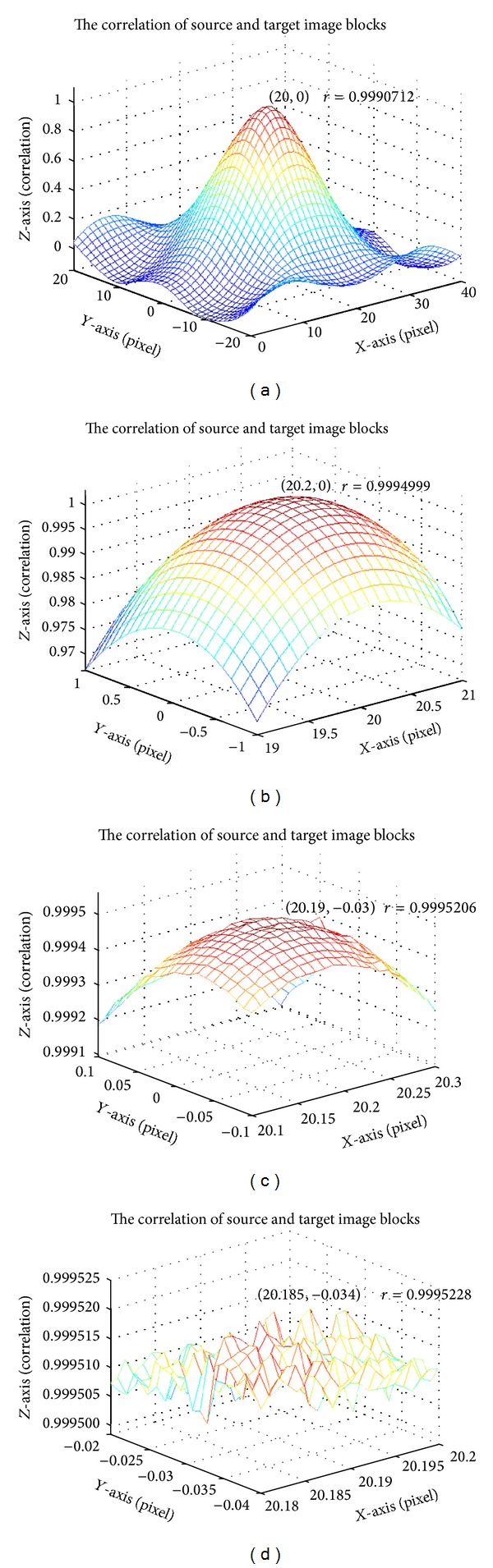
Variation of correlation coefficient with displacement scales: (a) 1 pixel, (b) 0.1 pixel, (c) 0.01 pixel, and (d) 0.001 pixel.

After subpixel offsets, the estimation block can be calculated with the correlation coefficient to compare with a previous maximal correlation coefficient, allowing us to evaluate the adaptive size of pattern in terms of subpixel accuracy. Because the gray pixel value ranges from 0 to 255 in this work, the precision of 0.1 pixel value is acceptable. The precision may be unreliable for a subpixel value less than 0.01.  Figure 7 shows the variation of a correlation in different pixel scales. In too small of a scale, the correlation is too sensitive to affirm the reliability of the displacement. In the subpixel analysis of this work, the divided factor can determine the subpixel scale. For instance, the factor is set to 10, indicating one pixel divided into ten 0.1 subpixels. The 0.1 pixel is divided into ten 0.01 subpixels.

**Figure 8:**
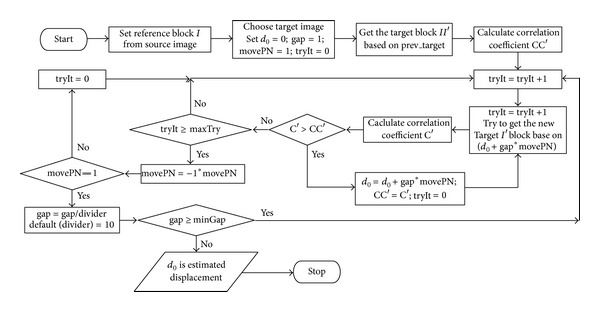
Flowchart of displacement evaluation algorithm, which has better execution time and accuracy than that in our earlier work.

Another important algorithm, displacement evaluation algorithm, compares the source image block and target image block by using the digital image correlation coefficient to evaluate the displacement of pattern. Based on a fixed attempt, our previous study evaluated displacement using digital image correlation for different pixel scales [12]. Additional computing time is expended in evaluating the iteration. Therefore, an improved algorithm (Figure 8) shows the flowchart. Based on the location and size of the pattern in a source image estimated from a previous algorithm, a source image block *I* is created as a reference block. Some image blocks are selected from a target image to compare with reference block *I*. Displacement of the target image is determined by the maximal correlation coefficient of these image blocks with reference block *I*. Parameters *d*_0_, gap, movePN and tryIt are initialized as given value. The *d*_0_ value is the currently estimated displacement; the gap is a moving step with a pixel unit; movePN is the moving direction where 1 denotes positive direction and −1 denotes a negative one; tryIt is the count of an estimated fail in a particular direction. An estimated fail indicates that the current correlation coefficient is less than the current maximal correlation coefficient. The first target image block was selected based on the previous displacement. A closer target block to the pattern of a target image generally implies a larger correlation coefficient of the target and reference block. The algorithm attempts bilaterally to obtain the maximal correlation coefficient. In our previous work [12], the measurement system has a fixed number of attempts to evaluate the correlation in a positive or negative direction. Several computing times were wasted in iterative process. The new proposed algorithm can quickly obtain the maximal correlation coefficient to determine the evaluated displacement of the target image. A flexible number of attempts (maxTry) can be assigned to the measurement system by a user or default. Analysis results indicate that an attempt can still be made to estimate the accuracy displacement in a slight variation of source and target image. The above algorithms are implemented using MATLAB software.

Images are acquired from a high-speed digital camera by using LabVIEW software. However, the image acquisition program encounters two problems: the total image storage capacity is too large and too high of a sampling rate causes an insufficient computational time. As the first problem, the program cuts the image into a smaller critical block to save the image. The critical block includes the pattern and approximate number of maximum pixel displacements. Moreover, the program records the number of each image frame to identify the lost frame in the acquirement procedure of a higher sampling rate. Based on the interpolation method, the images are reconstructed to increase the accuracy of time-history data.

**Figure 9:**
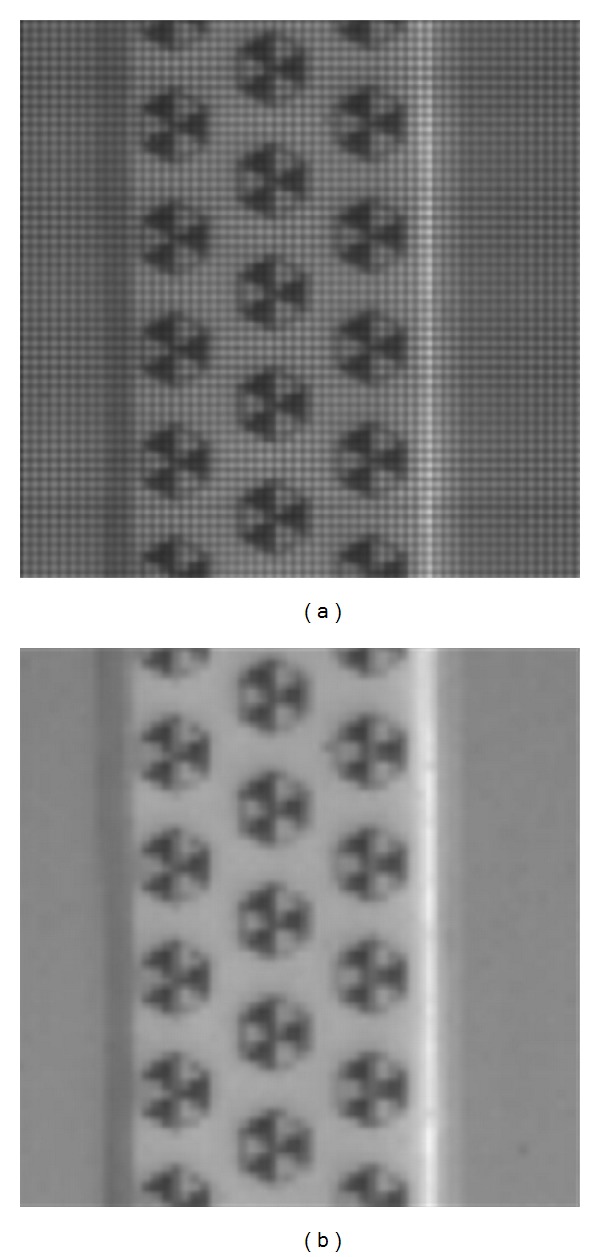
Bayer decoding. (a) Original captured image frame with many grid lines and (b) Bayer-decoded image.

The digital camera, which has a Bayer filter sensor type, must decode an image for the Bayer pattern. Otherwise, the image displays many grid lines (Figure 9). This problem can be solved with a library function of LabVIEW.

The original length unit of image displacement is integer pixel size. The actual length (*L*) of the pattern is a known quantity, and the pixel width (*W*) of the pattern has been evaluated by the measurement system. The actual structural displacement can be calculated using estimated pixel displacement to multiply by a pixel ratio (*R*_*p*_) from (5). At this point, the time-history displacements are estimated completely in the digital image measurement system:

(5) $$\begin{array}{c} R_{p}=\frac{L}{W},\;\;\left(u,v\right)=\left(d_{x},d_{y}\right)*R_{p}. \end{array}$$

## 4. Results and Analysis

### 4.1. Numerical Simulation Result

**Figure 10:**
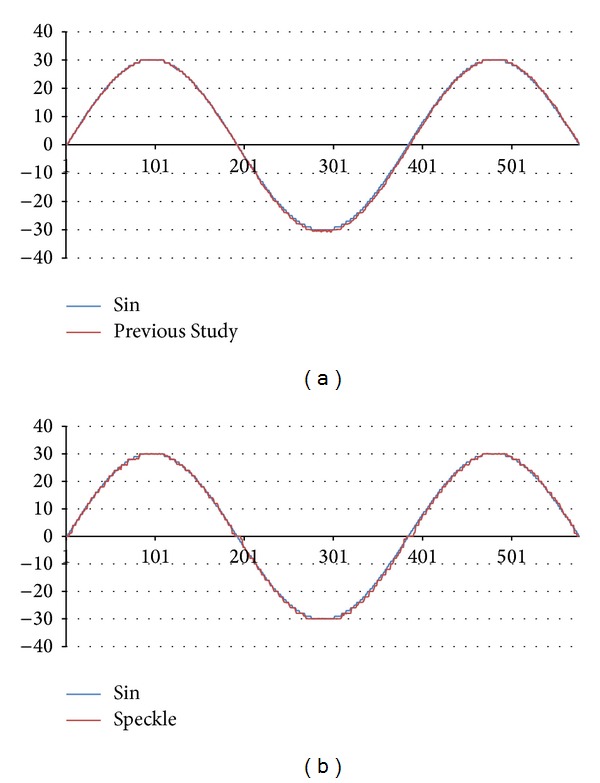
Numerically simulated time history of displacement. (a) Hive triangle pattern and (b) random speckle.

The work developed a numerical simulated image, which included digital random speckle and hive triangle patterns, as shown in Figure 3. The simulated image was generated from the PHP web language. A FLASH animation program was designed to display the simulated image in different locations with sine variation. The animation was played on a computer monitor, and the time-history displacement was estimated in the measurement system.  Figure 10 summarizes those results.

The time-history displacement calculated by using the proposed hive triangle pattern was smoother than that calculated by using a digital random speckle. The example considered in this work indicated that the result of using hive triangle pattern was better than the digital random speckle to compare with simulated sin displacement. Speckle pattern cannot be accurately identified with poor image quality. In addition, this is owing to the fact that the position and size of an image block affected the computational time and accuracy of evaluation in to digital image correlation method. Therefore, several evaluations were estimated and compared within different positions and sizes of the image block.  Table 1 summarizes those comparison results. The block of case 1 is selected in human visual discrimination, and case 2 is chose by the measurement system. Case 3 is based on case 2, with the image block size cropped from 57 × 49 to 49 × 42. Simulation results reveal that the execution time is shorter but the root mean square error (RMSE) reveals that the accuracy is lower than in case 2. Cases 5 and 6 involve the random speckle pattern with different block sizes. The block in case 7, as in case 2, includes one-hive triangle pattern which is detected by the measurement system, and case 8 involves parts of three-hive triangle patterns. “Time” represents the execution time in any case. For a given block, the execution time herein is shorter than in the previous study. The faster execution supports applications that are closer real-time systems. The RMSE herein is better than that achieved in the aforementioned previous study. Simulation results reveal that the work herein is better than that in the previous study in terms of execution time and accuracy.

For the random speckle pattern, a higher RMSE appeared in which an image block is located in the interior region of the entire speckle. When the width of a random speckle image block is greater than that of the entire speckle, a satisfactory accuracy can be obtained. Outside of the entire speckle is white; the boundary is thus significantly different from inside to outside. Closely examining the acquired images revealed that the image quality was unacceptable, owing to an insufficient exposure time and highly compact density of the random speckle. In displacement estimation, the correlation coefficient of speckle pattern cannot be correctly estimated, owing to undesirable image quality. However, in the ideal simulated images, the results closely correspond to the actual displacement during simulation. The theory of estimated displacement with speckle is valid, only limited under certain circumstances. Based on our experimental results, we recommend increasing the speckle size and spacing. Additionally, the entire speckle region is set as the reference image block is considered another possible solution. The additional computational time cost must be spent for a larger image block.

This work improves the ability of the proposed algorithm used to estimate the displacement in the previous study.  Table 1 indicates that the proposed approach is better than the previous algorithm within an identical position and size of image block including RMSE and frequency peaks. In particular, displacement estimation can obtain more accurate results by using the pattern location algorithm to locate the adaptive reference image block. Despite only a slight improvement, the findings still demonstrate the advantages of the proposed algorithm. Therefore, the algorithm is a feasible noncontact displacement estimation method, with subsequent experiments undertaken using this method.

### 4.2. Simulation Results of a Small Earthquake

**Figure 11:**
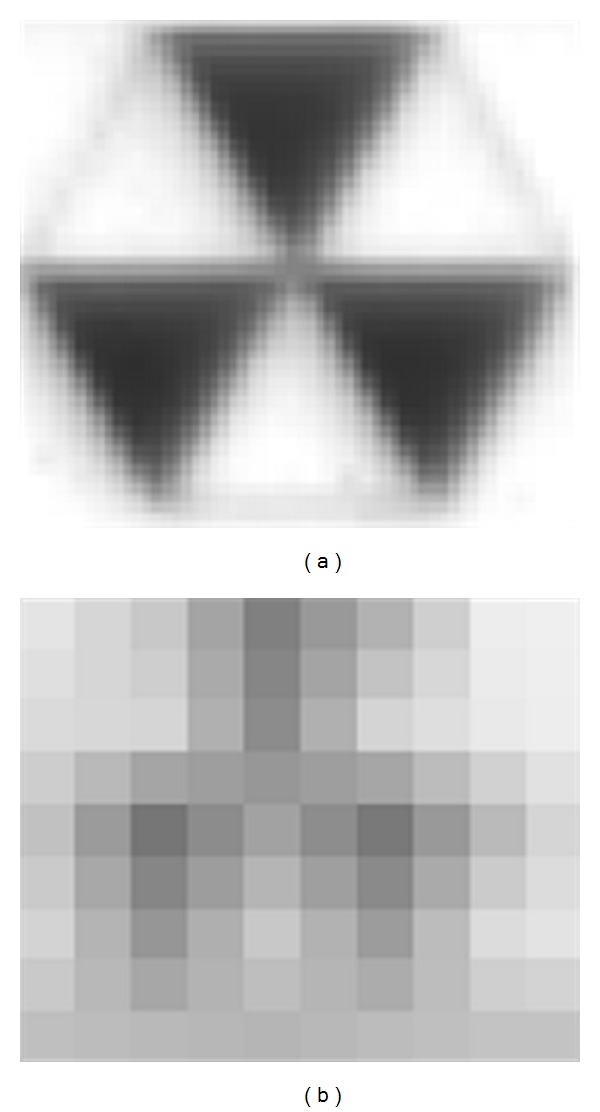
Patterns with different resolutions. (a) High resolution, 57 × 49. (b) Low resolution, 10 × 9.

**Figure 12:**
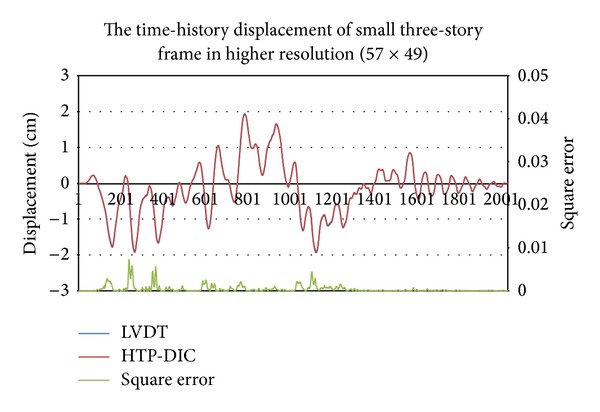
Time history of displacement and square error of displacement of small three-story frame at high resolution (57 × 49).

**Figure 13:**
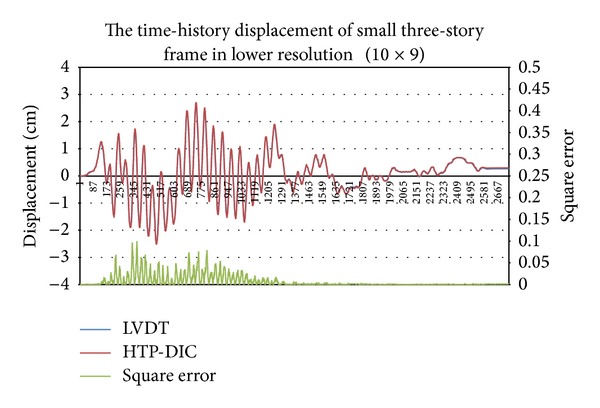
Time history of displacement and square error of displacement of small three-story frame at low resolution (10 × 9).

The experiment involves two image resolutions.  Figure 11 shows the pattern image within different resolutions. First, the camera lens focuses on the second floor in a large resolution. Figure 2 shows the sample of a captured image, while Figure 12 shows the estimated time-history displacement and square error. The curve of LVDT is very similar to the estimation of a measurement system. The peak value of frequency calculated in FFT is 0.1258944579 and 0.1259031566, respectively. The relative error of a frequency is −6.90952E-05 and the root mean square error is 0.020523298. Second, the camera records the entire three-story frame building. Figure 4 shows the sample of a captured image. The El Centro earthquake is generated from the small shaker table, and the time-history displacement is estimated from the digital image measurement system.  Figure 13 summarizes those results, indicating that the curves are similar to each other. The peak value of frequency calculated in FFT is 1.835178054 and 1.835320253, respectively. The relative error of a frequency is −7.74851E-05. Two peak values of frequency are similar to identical. The root mean square error is 0.084863876. Owing to lower image resolution, in the displacement estimation, the results cannot be estimated accurately. In sum, the curve of time-history displacement resembles that of the results of LVDT.

### 4.3. Data Analysis

This work extends the results of our previous study [12]. In the evaluated iteration, a fixed number of attempts which attempt to derive the maximal correlation coefficient are transformed into flexible maximal attempts. The computational time in this work is significantly decreased over that that in our previous study.  Table 1 shows the execution time in different experiments. The position and size of the image block directly affect the computational time of the estimation and the accuracy of the results. The pattern location algorithm of the measurement system can precisely identify the position and size of the pattern for the reference image block. By using the reference image block, calculating the correlation coefficient can increase the accuracy of estimated displacement. However, this pattern location algorithm also has certain limitations. When the resolution of an acquired image is insufficiently high, the position and size of the pattern cannot be located correctly. For instance, the width of a pattern is only 10 pixels in an acquired image; in addition, the position and size of pattern are still difficult to find. The width of a pattern is 57 pixels in the first experiment of Section 4.2, while the position and size of pattern can be obtained with this algorithm.

The reference image block is set based on a visual observation to determine the position and size of pattern in our previous work. The estimated displacement is similar to the measurement of LVDT. In contrast, in this work, the reference image block set accurately can achieve more precise results. Actually, the acquired image has some reference objects, which can be used as a reference image blocks to estimate the structural displacement. However, the results may not be satisfactorily accurate.

## 5. Conclusion

This work presents a novel digital image measurement system, which is a feasible option for noncontact measurement. The time-history displacement estimated by the digital image system is very similar to LVDT in some experiments. The curves shown in Figure 12 nearly overlap each other in all periods. The curves shown in Figure 13 have a similar trend yet are inaccurate. Additionally, the actual length of one pixel is presented in the pixel ratio *R*_*p*_. If *R*_*p*_ is large, the image is rough; if *R*_*p*_ is small, the image is meticulous. By using subpixel analysis, the measurement system increases the accuracy to a pixel value of 0.1, or even 0.01. Therefore, if *R*_*p*_ is less than 0.1 mm, the accuracy easily achieves 0.01 mm. Based on our results, we conclude the following:

- in a higher resolution, the time-history displacement shows highly similar results. In a lower resolution, the displacement trend is approximate, yet the error of displacement is too large. The digital image measurement system is applicable in estimating the displacement of structural test in a higher resolution,
- the higher sampling rate decreases the exposure time of the camera to affect the images quality, in which the image acquisition program does not fully access all images,
- the setup of the image measurement system is relatively easy, as well as a very serviceable scheme, and
- the size of hive triangle pattern might cause an error in the measurement procedure and, therefore, several patterns are measured to diminish the error.

## Highlights

- The setup of the image measurement system is relatively easy.
- High-speed digital camera is suitable in experimental measurement.
- A specific hive triangle pattern is proposed.
- An evaluation of peak of frequency is accurate with digital image correlation.
- Numerical and experimental studies reveal the feasibility of proposed measurement system.

## Figures and Tables

**Table 1:** The comparison of numerical simulation result.

	Simulation case	Block^a^	Execution time (s)	RMSE
Previous study [12]	1	64 × 280_30 × 30	26	0.595849723
	2	51 × 271_57 × 49	81	0.571394047
	3	55 × 275_49 × 42	64	0.590442405
	4	51 × 271_205 × 10	49	0.572254826
	5	160 × 120_120 × 30	76	0.726009534
	6	70 × 50_205 × 10	46	0.68851133

This work	7	51 × 271_57 × 49	16	0.554749224
	8	51 × 271_205 × 10	13	0.550491283

^a^The datum denotes (Left) × (Top)_(Width) × (Height).

## References

[1] Çelebi M (2002). Seismic instrumentation of buildings (with emphasis on Federal buildings).

[2] Lin TH, Hung SL, Huang CS, Lin TK (2012). Detection of damage location using a novel substructure- based frequency response function approach with a wireless sensing system. *International Journal of Structural Stability and Dynamics*.

[3] Peters WH, Ranson WF (1982). Digital imaging techniques in experimental stress analysis. *Optical Engineering*.

[4] Chu TC, Ranson WF, Sutton MA (1985). Applications of digital-image-correlation techniques to experimental mechanics. *Experimental Mechanics*.

[5] Pan B, Wang Q (2013). Single-camera microscopic stereo digital image correlation using a diffraction grating. *Optics Express*.

[6] Amodio D, Broggiato GB, Campana F, Newaz GM (2003). Digital speckle correlation for strain measurement by image analysis. *Experimental Mechanics*.

[7] Chung H-C, Liang J, Kushiyama S, Shinozuka M (2004). Digital image processing for non-linear system identification. *International Journal of Non-Linear Mechanics*.

[8] Shih M-H, Sung W-P, Tung S-H, Bacinskas D, Kaklauskas G (2011). Developing three-dimensional digital image correlation techniques to detect the surface smoothness of construction materials. *International Journal of Materials and Product Technology*.

[9] Shih MH, Sung WP, Chen SC (2012). Application of digital image correlation technique to monitor dynamic response of building under earthquake excitation. *Advanced Science Letters*.

[10] Schreier HW, Braasch JR, Sutton MA (2000). Systematic errors in digital image correlation caused by intensity interpolation. *Optical Engineering*.

[11] Sutton MA, Orteu JJ, Schreier HW (2009). *Image Correlation for Shape, Motion and Deformation Measurements*.

[12] Hung SL, Lu YC, Tizani W The study of combining hive-grid target with sub-pixel analysis for measurement of structural experiment.

